# iNOS-Produced Nitric Oxide from Cancer Cells as an Intermediate of Stemness Regulation by PARP-1 in Colorectal Cancer

**DOI:** 10.3390/biom15010125

**Published:** 2025-01-14

**Authors:** María del Moral-Martinez, Paula Sánchez-Uceta, Ruben Clemente-Gonzalez, Sara Moreno-SanJuan, Jose D. Puentes-Pardo, Huda Khaldy, David Lopez-Perez, Javier Arnedo, Jorge Casado, Luis Martínez-Heredia, Angel Carazo, Josefa León

**Affiliations:** 1 Unidad de Gestión Clínica de Aparato Digestivo, Hospital Universitario Virgen de las Nieves, 18014 Granada, Spain; 2 Instituto de Investigación Biosanitaria de Granada (ibs.GRANADA), 18012 Granada, Spain; 3 Servicio de Microscopía y Citometría, Instituto de Investigación Biosanitaria de Granada (ibs.GRANADA), 18012 Granada, Spain; 4 Servicio de Biología Fundamental, Centro de Instrumentación Científica, Universidad de Granada, 18071 Granada, Spain; 5 Laboratory of Cell Biology, National Cancer Institute, National Institutes of Health, Bethesda, MD 20892-4258, USA; 6 Departamento de Estadística e Investigación Operativa, Universidad de Granada, 18071 Granada, Spain; 7 Instituto de Salud Carlos III, CIBER de Fragilidad y Envejecimiento Saludable (CIBERFES), 28029 Madrid, Spain; 8 Unidad de Gestión de Microbiología, Hospital Universitario San Cecilio de Granada, 18016 Granada, Spain; 9 Unidad de Gestión Clínica de Aparato Digestivo, Hospital Clínico Universitario San Cecilio, 18016 Granada, Spain

**Keywords:** colorectal cancer (CRC), PARP-1, iNOS, cancer stem cells (CSCs)

## Abstract

PARP-1 has been linked to the progression of several types of cancer. We have recently reported that PARP-1 influences tumor progression in CRC through the regulation of CSCs in a p53-dependent manner. In this study, we propose that nitric oxide (NO) produced by inducible nitric oxide synthase (iNOS) could act as a mediator. We evaluated the expression of iNOS in a cohort of patients previously used to analyze the effects of PARP-1 on CRC in relation to p53 status. We also developed an in vitro model in which PARP-1 was stably overexpressed. In CRC patients, iNOS expression correlated with the differentiation grade, and with a high expression of CSC markers, although only in wild-type p53 tumors, as previously found for PARP-1. In vitro, overexpression of PARP-1 induced increased growth and stemness in wild-type p53 cells, while exerting the opposite effect on mutated ones, as expected. Treatment with 1400 W, a selective inhibitor of iNOS, or gene silencing of the gene counteracted the effects of PARP-1 in both p53 wild-type and p53 mutated cells. Given that the development of resistance has been demonstrated after treatment with PARP-1 inhibitors, iNOS could be considered a new therapeutic target in CRC, although only in patients with wild-type p53 tumors.

## 1. Introduction

Colorectal cancer (CRC) is a very common cancer worldwide and the second leading cause of cancer death [1]. Surgery and chemotherapy are the primary treatment modalities for CRC [2]. However, the response to chemotherapy and the development of resistance show large heterogeneity among patients, even at the same stage of the disease, which has been associated with the existence of cancer stem cells (CSCs) [3,4].

CSCs are a group of cells in a tumor organized in a hierarchical manner, characterized by self-renewal and pluripotency [5]. Their existence has been identified in almost all types of human cancers [6]. They can differentiate into various cancer cell phenotypes and maintain their population through interactions with the microenvironment [5]. CSCs are responsible for tumor initiation, tumor regeneration capacity after treatment, and therapy resistance [5]. Therefore, exploring the factors affecting CSC functions will improve therapeutic efficacy and outcomes of cancer patients.

DNA damage induction is the main mechanism of chemotherapeutic drugs used in CRC [7]. CSCs have an elevated capacity to repair chemotherapy-induced DNA breaks, which highly depend on poly(ADP-ribose) polymerase-1 (PARP-1) [8]. In fact, PARP-1 expression is not homogeneous in tumor cells, but appears to be higher in cells with CSC characteristics [8,9,10]. In addition, PARP-1 has been shown to contribute to CSCs’ survival, renewal, tumor0initiating properties, and resistance to therapy [11]. All these reports led to the therapeutic targeting of PARP-1 in CSCs as an efficient strategy to treat cancer. Thus, PARP-1 inhibitors in single or combined therapy have been used in different types of cancer to eliminate this subpopulation of cells [12]. In this sense, although clinical trials carried out in breast and ovarian cancer have yielded positive results, this has not been the case in CRC [13].

Very recently, our research group described a dual role of PARP-1 in the regulation of CSC phenotypes in CRC. PARP-1 overexpression increases stemness in p53 wild-type cells, while the opposite effect was found in cells harboring mutated p53. These results correlated with those obtained in patients, in which high PARP-1 expression was found to be an independent prognostic factor for survival in CRC, but only in patients with mutant p53 [14]. However, the mechanism implicated in this effect is still unknown.

Although most studies have focused on the role of PARP-1 in DNA repair, the functions of PARP-1 have extended to transcription, metabolic regulation, cell death, and inflammation, among others [15]. The activation of PARP-1 plays an important role in the up-regulation of inflammatory signaling [16,17,18,19,20]. In this regard, PARP-1 has been described as an activator of the inducible nitric oxide synthase (iNOS) through regulation of the NF-κβ transcription factor [21], and also by direct binding to the iNOS promoter [22]. The iNOS enzyme (also called NOS2) is one of the isoforms responsible for the synthesis of nitric oxide (NO), a free radical involved in physiological processes such as vasodilation, neurotransmission, platelet aggregation, inflammation, and macrophage-mediated immunity, although it has also been implicated in several pathologies [23]. Interestingly, overexpression of iNOS can limit PARP-1 DNA binding activity and the ability to transactivate the iNOS promoter. This feedback mechanism can limit the excessive NO generation and subsequent cell death observed in inflammation or stroke [22].

The expression of iNOS is high in cancer, particularly in CRC [24], both in premalignant lesions [25] and in adenocarcinoma [26]. In terms of location, the overexpression of iNOS has been found in cancer cells and in the immune system cells that infiltrate the tumor [25,26]. Recent reports indicate that iNOS/NO can generate and maintain CSCs [27]. NO synthetized by iNOS has been implicated in the initiation of carcinogenesis in CRC through neoplastic transformation of the intestinal stem cells at the bottom of the crypts [28] and in the regulation of the stemness properties of CSCs through a variety of signaling pathways [29]. In addition, clones of CSCs with high endogenous NO production exhibited higher tumorigenicity capacity, which could be used from a therapeutic point of view [29].

However, the effects of NO on either promoting or inhibiting carcinogenesis and tumor growth depend on the origin of the tumor, the stage of cancer, and the cell types in the tumor microenvironment [30], highlighting a complex role of NO in cancer. In fact, it has been proven that the inhibition of NO production by iNOS can promote metastasis in CRC [31], while opposite results were showed by other authors [32]. Therefore, the main objective of this study is to clarify the role of iNOS/NO in CRC, by studying whether iNOS acts as an intermediate in the ability of PARP-1 to regulate the phenotype of CSCs.

## 2. Materials and Methods

### 2.1. Patients

The Ethical Committee of Clinical Research of Granada (project code: PI-067/2013; date of approval: 24 January 2014) approved this study. This research is part of a larger prospective study in which 201 patients were initially recruited [14]. In this case, we included 186 samples from patients who underwent surgery for primary sporadic CRC, which were provided by the Andalusian Tumor Bank Network (RBTA) (Table S1). The inclusion criteria comprised people over 18 years, without hereditary burden, not treated with neoadjuvant therapy, and not previously diagnosed or treated for cancer. All of them gave written informed consent for the use of samples in biomedical research. Immediately after the samples were obtained, viable tumor tissues and adjacent normal mucosa were dissected and fresh-frozen in Tissue-Tek1 (Optimal Cutting Temperature Compound, Sakura Finetek Europe B.V., Zoeterwoude, The Netherland).

### 2.2. Analysis of p53 Mutations in CRC Samples

First, the extraction of genomic DNA from tissues was performed using a QIAamp DNA Mini Kit (Qiagen, Hildem, Germany), following the commercial indications. Quantification of DNA was performed in a NanoDrop ND-1000 (Implen GmbH, Munich, Germany), and its integrity was assessed by electrophoresis on an agarose gel. TP53 mutations in exons 2–10 of the tissues were analyzed by PCR, using specific primers (Table S2) [33]. PCR products were purified using Wizard SV gel and PCR clean-up system (Promega, Madison, WI, USA). PCR products were sequenced using the 3130 XL Apllied Biosystems, Foster City, CA, USA). The results were analyzed with the Chromas Lite 2.1.1 (St South Brisbane, QLD, Australia) software [16].

### 2.3. RNA Extraction and First-Strand cDNA Synthesis

Total RNA from tissues or cultured cells was obtained using TRIzol reagent (Invitrogen, Life Technologies, Carlsbad, CA, USA). The quantity of total RNA was determined by UV spectrophotometry, and its integrity was assessed by agarose gel electrophoresis. First-strand cDNA was synthetized using the qScript™ cDNA Synthesis kit (Quanta Biosciences, Gaithersburg, MD, USA).

### 2.4. Real-Time PCR (RT-PCR)

cDNA was amplified with the PerfeCTa SYBR Green SuperMix Kit (Quantabio, Beverly, MA, USA), using specific primers for PARP-1, iNOS, CD44, CD133, UBC, TBP, and RPS13 (Table S3). UBC, TBP, and RPS13 were used as housekeeping genes. Standard curves representing Ct values versus log cDNA dilution were constructed for each gene.

### 2.5. Cell Culture and Reagents

Two CRC cell lines obtained from the American Type Culture Collection (ATCC, Rockville, MD, USA) with different p53 status were used in this study: HCT-116 (p53 wild-type) and HT-29 (p53 mutated). Both types were cultured in DMEM (Gibco, Carlsbad, CA, USA) supplemented with 2 mM L-glutamine, 10% FBS, and a 1% antibiotic–antimycotic cocktail containing penicillin (100 U/mL), streptomycin (100 µg/mL), and amphotericin B (250 ng/mL) (Gibco, Carlsbad, CA, USA) at 37 °C with 5% CO_2_.

Further, 1400 W was purchased from Selleckchem (Houston, TX, USA). It was diluted in PBS at a concentration of 20 mM and conserved, frozen at −20 °C until use.

### 2.6. Transfection Protocols

To obtain stable cell clones overexpressing PARP-1, 150,000 cells per well were seeded in a 6-well plate, maintained overnight, and transfected with lipofectamine 2000 transfection reagent (Thermo Fisher Scientific, Waltham, MA, USA) according to the manufacturer’s instructions. We used a DNA to lipofectamine ratio of 1:2.5 (*w*/*v*). After 24 h, the medium was changed, and cells were selected by adding 1.5 mg/mL (final concentration) of G418. Medium was changed every two days until the colonies formed were visible. Then, they were passed individually to a new plate, left to growth using 0.4 mg/mL (final concentration) of G418, and finally tested by Western blotting. Plasmid expression vector pCMV6 containing the human PARP-1 gene and the corresponding empty vector were obtained from Origene Technologies (Rockville, MA, USA).

Once the overexpression of PARP-1 was confirmed, inducible NOS (iNOS) was inhibited by transient transfection with siRNA-iNOS (siNOS2) (Santa Cruz Biotechnology Inc., Dallas, TX, USA) (50 ng/mL) and lipofectamine 2000 transfection reagent (Thermo Fisher Scientific, Waltham, MA, USA), according to the manufacturer’s instructions. Scrambled siRNA was used as negative control. In this case, 100,000 cells were seeded in a 6-well plate, left to attach overnight, and transfected. Then, the medium was changed and maintained for 72 h more to test the iNOS silencing by using Western blotting.

### 2.7. Immunoblotting

Stable clones overexpressing PARP-1 were transfected with siRNA-iNOS (si-NOS2), and collected after 72 h or treated with 1400 W 20 µM for 72 h. After the treatments, the cells were washed in ice-cold PBS and incubated in RIPA buffer containing protease inhibitors. Then, 35 µg of proteins were transferred to PVDF membranes and probed with an appropriate antibody raised against PARP-1, which recognizes both the full (116 kDa) and cleaved (85 kDa) forms of the protein (Abcam, Cambridge, UK), iNOS (Proteintech, Rosemont, IL, USA), and one against β-actin (Santa Cruz Biotechnology, Dallas, TX, USA). Secondary antibodies were visualized by enhanced chemiluminescence using HRP-conjugated secondary antibodies (Santa Cruz Biotechnology). Band intensity was quantified using Quantity One 4.6.8 (Bio-Rad Laboratories, Inc., Hercules, CA, USA) software.

### 2.8. Nitrites Assay

Cells were seeded in 96-well plates at a density of 4000 cells per well, and transfected with siNOS2 during 72 h or treated with 1400 W 20 µM during 72 h. Then, 100 µL of supernatants was assayed with the nitrate/nitrite fluorimetric kit to determine total nitrites (Cayman Chemical Company, Ann Arbor, MI, USA). Fluorescence was measured in a microplate reader (TRIAD series, Dynex Technologies Multimode Reader, Chantilly, VA, USA). All experiments were performed three times in duplicate.

### 2.9. MTT Assay

Cells were seeded in 96-well plates at a density of 4000 cells per well, and transfected with siNOS2 during 72 h or treated with 1400 W 20 µM during 72 h. Then, 50 µg of MTT (stock solution 5 mg/mL of Dulbecco’s PBS) was added to each well over 4 h. A volume of 100 μL lysis buffer (20% SDS in 50% N, N-dimethylformamide at pH 4.7) was added and the cells were incubated at 37 °C overnight. Absorbance was measured on a microplate reader (TRIAD series, Dynex Technologies Multimode Reader) at 570 nm. All experiments were performed three times in quadruplicate.

### 2.10. Apoptosis Assay

Apoptosis was analyzed using the IP-Annexin V kit (BD Biosciences, Berkshire, UK). Briefly, cells were seeded in 6-well plates and after treatment with 1400 W 20 µM or transfection with siNOS2 during 72 h, they were trypsinized, washed twice with cold PBS, and resuspended in 1X Binding Buffer. Then, 10^5^ cells were incubated with 5 µL of FITC Annexin V and 5 µL PI and incubated for 15 min at room temperature in the dark. Finally, 400 µL of 1X Binding Buffer were added to each tube. Cells were analyzed by flow cytometry within 1 h using the BD FACS Aria IIIu Flow Cytometer (Becton Dickinson, BD Bioscience, UK).

### 2.11. Clonogenic Assay

Cells were treated with 1400 W 20 µM 24 h or transfected with siNOS2 and left to grow for 24 h. Then, cells were seeded in 6 well-plates at a concentration of 1000 cells/well and left to grow for 14 days. The medium was removed, and the cells were washed with PBS and incubated for 5 min with 0.5% oxalate crystal violet solution in methanol 50%. Colonies greater than 50 cells were counted.

### 2.12. Aldefluor Assay and Cell Surface Markers Analysis

ALDH1 activity was detected using the Aldefluor assay (Stem Cell Technologies, Vancouver, BC, Canada) kit. After treatment with 1400 W or transfection with siNOS2 during 72 h, cells were collected, suspended in Aldelfuor assay buffer containing ALDH1 substrate (BAAA, 1 μmol/L per 1 × 10^6^ cells), and incubated for 45 min at 37 °C in darkness. The same quantity of cells was incubated with dethylaminobenzaldehyde (DEAB) to establish ALDH1 gates. The brightly fluorescent ALDH1-expressing cells were analyzed using BD FACSAria III flow cytometry (Becton Dickinson, BD Biosciences, UK).

To analyze the cell surface by flow cytometry using a BD FACSAria III (Becton Dickinson, BD Biosciences, UK), cells were incubated for 30 min in darkness at 4 °C with human anti-CD44-PE, anti-CD326-FITC, and anti-CD133-APC antibodies (Biolegend, San Diego, CA, USA).

### 2.13. Spheres Formation Assay

Stable clones overexpressing PARP-1 were transfected with siNOS2 or treated with 20 µM 1400 W over 24 h. After that, 1000 cells were resuspended in sphere culture medium (DMEM:F12, 1% penicillin/streptomycin; B27, 10 μg/mL ITS; 1 μg/mL hydrocortisone; 4 ng/mL heparin; 10 ng/mL EGF; 20 ng/mL FGF) in 96-well plates previously coated with poly-2-hydroxyethyl methacrylate (Merk, Darmstadt, Germany). Spheres greater than 75 μM in diameter were counted after 4 days by light microscopy.

### 2.14. Immunofluorescence Microscopy

Cells were plated on 24-well plates with coverslides, allowed to attach overnight, and treated with 1400 W 20 µM 72 h. After the treatments, the cells were fixed in ice-cold 4% formalin for 15 min, washed three times with 0.1% PBS-Tween 20, and incubated overnight at 4 °C with primary antibodies against PARP-1 (Abcam, Cambridge, UK) (1:100 dilution) and iNOS (Santa Cruz Biotechnology, Inc., Dallas, TX, USA) (1:50 dilution). Then, the cells were washed three times with 0.1% PBS-Tween 20 and incubated with secondary antibodies (Alexa Fluor 555-conjugated IgG; Alexa Fluor 488-conjugated IgG Molecular Probes, Eugene, OR, USA) for 1 h at room temperature. Nuclear staining was obtained by incubating the cells with Hoechst 3342 (Santa Cruz Biotechnology, Inc., Dallas, TX, USA). Negative controls (cells marked only with secondary antibodies) were performed to avoid background noise. Images were acquired using a ZEISS Celldiscoverer 7 microscope with Zen 3.3 BlueEdition software (Carl Zeiss Microscopy, GmbH, Oberkochen, Germany) and analyzed using Arivis Vision4D 4.1 software (Carl Zeiss Microscopy, GmbH, Germany), all of which were provided by the Scientific Instrumentation Centre at the University of Granada.

### 2.15. Statistical Analysis

Analysis of patient samples was performed using SPSS software version 15.0 for Windows (IBM, Chicago, IL, USA). Continuous variables were expressed using the median and interquartile range (IQR), while categorical variables were expressed as numbers and percentages. The mRNA levels of the adjacent non-tumor and tumor tissues were compared using the Wilcoxon *t*-test for paired samples. Next, analyses were performed by normalizing the mRNA levels of genes in tumor samples to the mRNA levels in non-tumor mucosa for each patient. The association of gene expression and clinicopathological characteristics was carried out using the non-parametric Kruskal–Wallis and Mann–Whitney U tests. Pearson’s test was used for correlation analysis after transforming the variables by applying natural logarithms. Fisher’s exact test was used to compare PARP-1 and iNOS levels and CSC markers. The high or low mRNA expression of each gene was determined based on the median of our study population. *p* values below 0.05 were considered significant, and confidence levels were set at 95%.

All the in vitro experiments were performed at least in triplicate and data were expressed as mean ± SD. After the normalization of variables, comparisons were performed using a *t*-test or 2 way-ANOVA with GraphPad Prism 7.0 (GraphPad, La Jolla, CA, USA) software.

## 3. Results

### 3.1. INos Expression Correlates with a Low Differentiation Grade in Tumor Tissues of CRC Patients

We measured iNOS mRNA expression in paired tumor and non-tumor tissues of a cohort of 186 CRC patients previously used to evaluate the prognostic implication of PARP-1 in CRC [14]. We found increased iNOS expression in the tumoral tissue compared to the paired non-tumoral mucosa in all patients (*p* < 0.0001) and in those harboring a wild-type p53 (*p* = 0.006), while no differences were found in cases with mutated p53 (*p* = 0.238). Considering PARP-1, its expression was found to be higher in tumor tissue than in adjacent non-tumor mucosa in all the cases studied (*p* < 0.0001), and in both wild-type p53 (*p* < 0.001) and mutated p53 (*p* < 0.013) (Table S4); these results are similar to those previously reported [14].

Next, we evaluated the involvement of iNOS in CRC progression. As shown in Table 1, iNOS expression increased in moderately and poorly differentiated versus well-differentiated tumors, although only in cases with wild-type p53. In addition, iNOS expression decreased as the size of the tumor increased in cases harboring wild-type p53. No relationship was found between iNOS expression and the other parameters studied, such as age, sex, tumor location (colon or rectum), number of affected lymph nodes, or pTNM stage. Considering PARP-1, we previously published that its expression correlates with the degree of tumor differentiation, including in wild-type p53 tumors [14]. These results were also found in the subcohort used for this study (Table S5).

Taking into account the above results, we analyzed the correlation between iNOS and PARP-1 expressions in our study cohort. The expression of both genes correlates in all cases (*p* = 0.0001), and regardless of p53 status (*p* = 0.002 and *p* < 0.0001 in wild-type p53 tumors and mutated p53 tumors, respectively) (Table 2).

### 3.2. iNOS Expression Is Related to Stemness Properties in Tumor Tissues of CRC Patients

**Figure 1:**
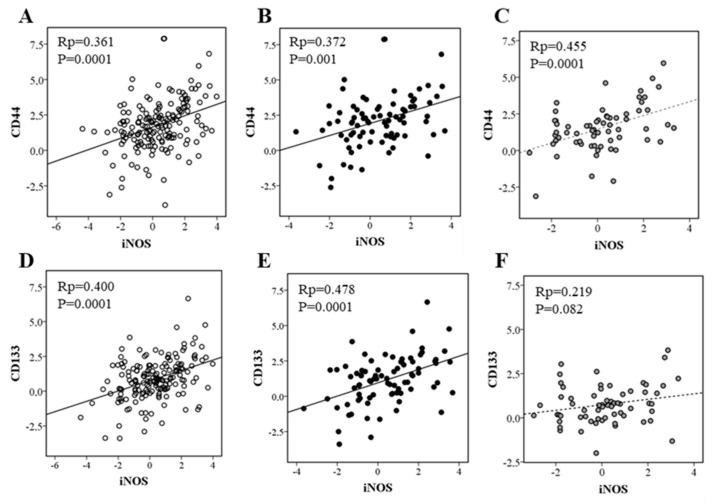
Correlation of iNOS mRNA expression and CD44 and CD133 mRNA expressions in (**A**,**D**) all cases; (**B**,**E**) wild-type p53 cases; (**C**,**F**) mutated p53 cases.

**Figure 2:**
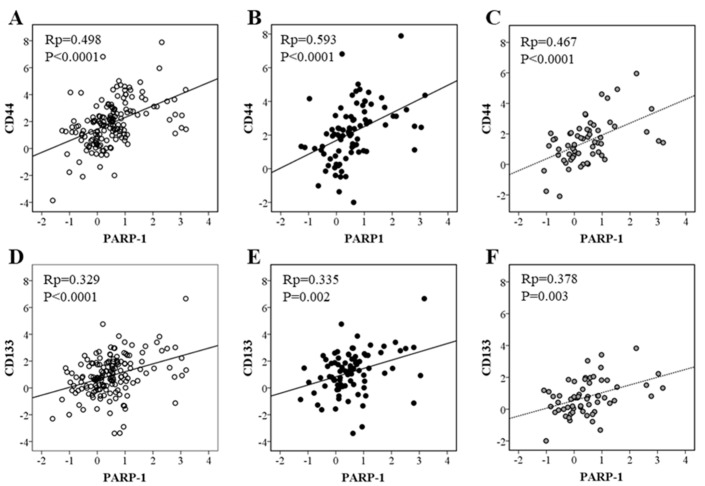
Correlation of PARP-1 mRNA expression and CD44 and CD133 mRNA expressions in (**A**,**D**) all cases; (**B**,**E**) wild-type p53 cases; (**C**,**F**) mutated p53 cases.

In the cohort of patients we included in this study, we also measured the expression of CD44 and CD133, two CSC surface markers described for CRC [5]. As shown in Figure 1, iNOS expression correlates with CD44 expression when considering all cases and regardless of the status of p53 (Figure 1A–C). On the other hand, iNOS and CD133 expressions correlated in all cases and in those harboring wild-type p53, but not in mutated p53 tumors (Figure 1D–F). PARP-1 expression correlated with both markers in all cases and regardless of p53 status (Figure 2).

**Figure 3:**
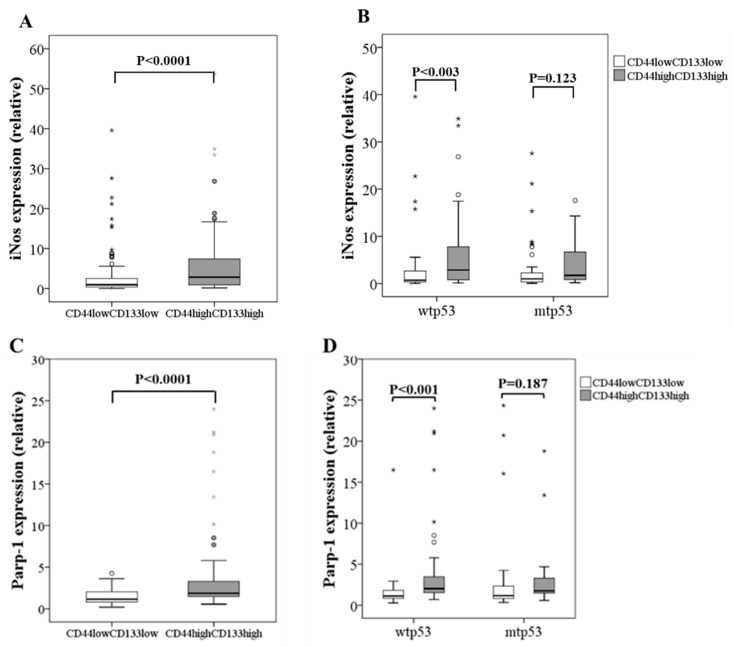
Relative mRNA expression of iNOS and PARP-1 in (**A**,**C**) all cases and (**B**,**D**) considering the status of p53 in CRC samples from patients considering the levels of CSC markers. Data represent the median and the interquartile range of the genes analyzed.

CSCs can be more accurately identified using a combination of more than one marker [5]. Therefore, and in order to more precisely study the relationship between iNOS and CSCs in the tumor, we have stratified the patients into CD133_low_CD44_low_ and CD133_high_CD44_high_. Interestingly, iNOS expression increases in CD133_high_CD44_high_ tumors (Figure 3A), mainly in those harboring wild-type p53 (Figure 3B). These results are similar to those found for PARP-1 in this subcohort (Figure 3C,D), and to those previously published by our group [14].

Next, we analyzed whether iNOS and PARP-1 expressions correlated in CRC tumors, considering the expression of CSC markers and p53. In cases harboring wild-type p53, iNOS and PARP-1 significantly correlated in CD133_low_CD44_low_ tumors and almost significantly correlated in CD133_high_CD44_high_ tumors. In cases with mutated p53, iNOS and PARP-1 significantly correlated in both CD133_low_CD44_low_ and CD133_high_CD44_high_ tumors. All the correlations found were also positive (Table 3).

### 3.3. Inhibition of NO Production by iNOS Reduced PARP-1-Induced Changes in Cell Growth and Viability in CRC In Vitro

**Figure 4:**
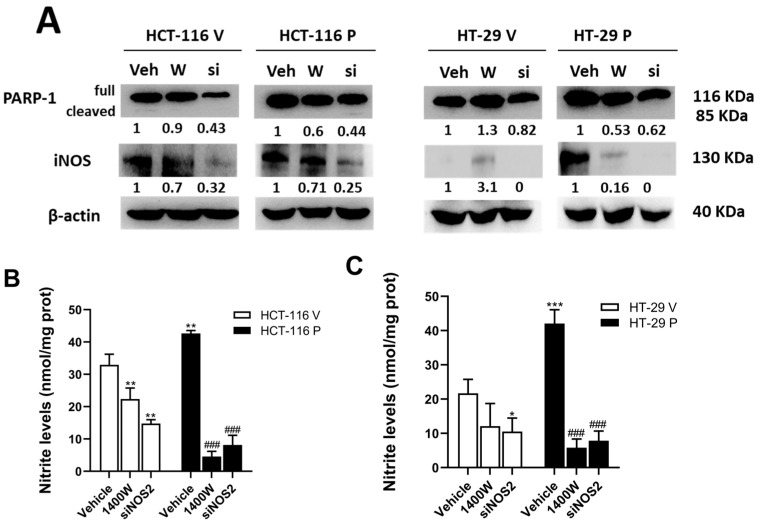
PARP-1 overexpression induces iNOS expression and NO production in CRC cells. (**A**) Protein expression of iNOS and PARP-1 in HCT-116 and HT-29 after PARP-1 overexpression (HCT-116 P and HT-29 P, respectively) and in mock transfected cells (HCT-116 V and HT-29 V, respectively). β-actin was used as a housekeeping gene. Veh: non-treated cells; W: treatment with 20 µM 1400 W; si: transfection with siNOS2. (**B**) iNOS activity, measured as nitrite levels in HCT-116 after PARP-1 overexpression (HCT-116 P) and in mock transfected cells (HCT-116 V). (**C**) iNOS activity, measured as nitrite levels in HT-29 cells after PARP-1 overexpression (HT-29 P) and in mock transfected cells (HT-29 V) *** *p* < 0.001 vs. HCT-116 V; ** *p* < 0.01 vs. HCT-116 V; * *p* < 0.05 vs. HCT-116 V; ^###^ *p* < 0.001 vs. HCT-116 P. Original Western blot images of Figure 4A can be found in Figure S2.

To further investigate whether NO synthesized from iNOS acts as an intermediate in the regulation of stem cell properties by PARP-1 in CRC, we used the pCMV6-PARP1 plasmid to stably overexpress PARP-1 in HCT-116 (p53 wild-type) and HT-29 (p53 mutated) cell lines. To obtain control cells, we used the empty plasmid pCMV6. Once the overexpression of PARP-1 was verified (Figure 4A), the stable clones (HCT-116 P and HT-29 P) and controls (HCT-116 V and HT-29 V) were transfected with a commercially available siRNA-iNOS/NOS2 (siNOS2) (see material and methods for details) to knock down iNOS gene expression and therefore to avoid iNOS protein synthesis and NO release (Figure 4B,C). As shown in Figure 4A, the overexpression of PARP-1 led to an increased expression of iNOS in both cell lines. As expected, the inhibition of iNOS expression (Figure 4A) and activity (Figure 4B) with siNOS2 resulted in a decrease in PARP-1 expression in the control and in cells overexpressing PARP-1. The treatment of cells with 1400 W 20 µM (a selective inhibitor of iNOS) inhibited NO production with an efficiency similar to that obtained after transfection with siNOS2 (Figure 4B). Cleaved PARP-1 (85 kDa) was detected in untreated (Veh) HCT-116 V and in HCT-116 P after transfection with siNOS2, although very weakly. This form of PARP-1 was not detected in HT-29-derived cells (Figure 4A).

**Figure 5:**
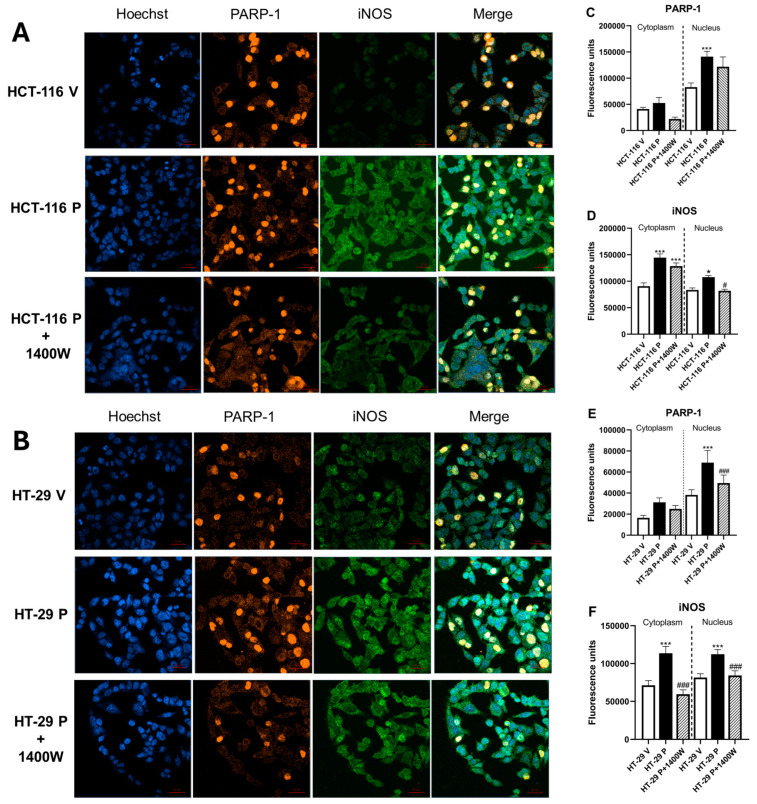
iNOS and PARP-1 proteins co-express in CRC cells. Controls and cells overexpressing PARP-1 from (**A**) HCT-116 and (**B**) HT-29 were seeded and only cells overexpressing PARP-1 were treated with either vehicle or 20 µM 1400 W over 72 h. Then, cells were immunostained with anti-iNOS antibody or anti-PARP-1 and visualized with a ZEISS Celldiscoverer 7 microscope, as described under Section 2. Images were analyzed with the Zen 3.3 (blue edition) software, obtaining fluorescence data on (**C**) PARP-1 and (**D**) iNOS, differentially in the cytoplasm and nucleus in HCT-116 V and HCT-116 P. Similarly, we obtained fluorescent data on (**E**) PARP-1 and (**F**) iNOS, differentially in the cytoplasm and nucleus in HT-29 V and HT-29 P. Data represent mean ± S.E.M of two experiments performed in duplicate. * *p* < 0.05 vs. V; *** *p* < 0.001 vs. V; ^#^ *p* < 0.05 vs. P; ^###^ *p* < 0.01 vs. P.

To analyze whether iNOS and PARP-1 co-express in the cells used in our in vitro model, we performed immunofluorescence assays in control cells (HCT-116 V and HT-29 V) and in cells overexpressing PARP-1 (HCT-116 P and HT-29 P) before and after treatment with 20 µM 1400 W. Our results revealed that both proteins co-expressed in the controls and in cells overexpressing PARP-1, regardless of treatment with the selective iNOS activity inhibitor and p53 status (Figure 5A,B).

After its overexpression, PARP-1 accumulated in the nucleus of both wild-type p53 and mutated p53 cells, while iNOS expression increased in the nucleus as well as in the cytoplasm, regardless of p53 status (Figure 5C,D).

**Figure 6:**
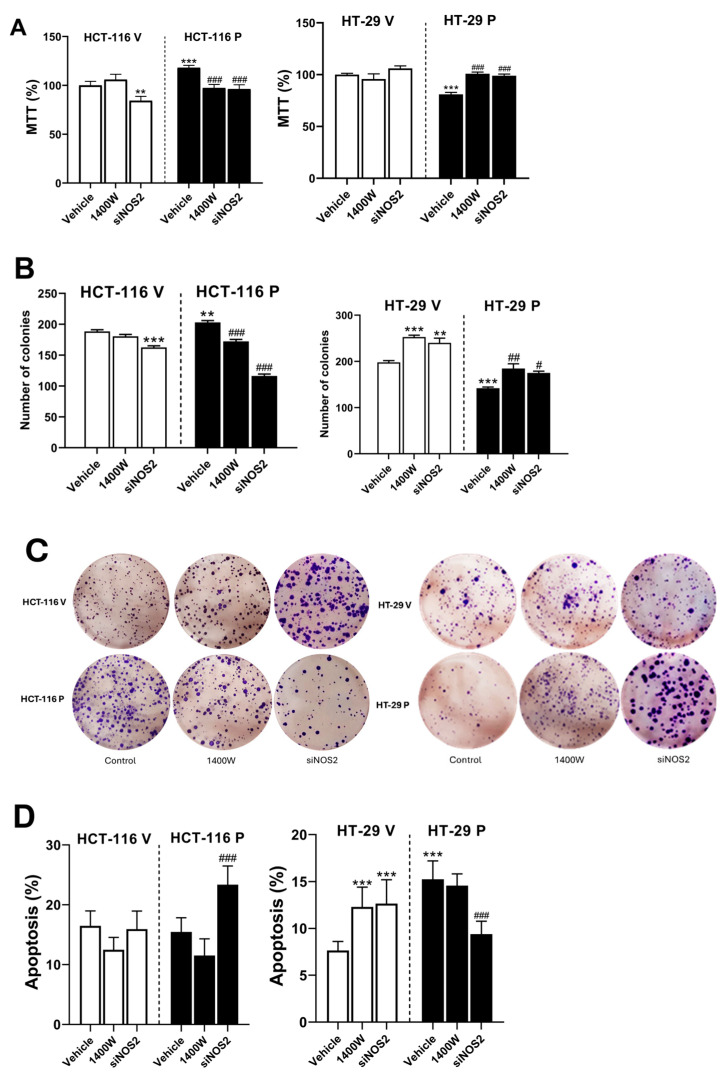
Regulation of cell growth and viability by iNOS in cells overexpressing PARP-1. (**A**) Cell growth of controls and cells -1 from HCT-116 and HT-29 overexpressing PARP, before and after treatment with vehicle, 20 µM 1400 W, or transfected with siNOS2, analyzed with the MTT assay. Data represent the mean ± SD. (**B**) Graphical representations of colony assay results of controls and cells from HCT-116 and HT-29 overexpressing PARP-1, before and after treatment with vehicle, 20 µM 1400 W, or transfected with siNOS2, as described in Section 2. Results are presented as means mean ± SD. (**C**) Representative example of colony formation assay. (**D**) Percentage of apoptosis of controls and cells from HCT-116 and HT-29 overexpressing PARP-1, before and after treatment with vehicle, 20 µM 1400 W, or transfected with siNOS2. Results are presented as means mean ± SD. ** *p* < 0.01 vs. HCT-116 V (vehicle) or HT-29 V (vehicle); *** *p* < 0.001 vs. HCT-116 V (vehicle) or HT-29 V (vehicle); ^#^ *p* < 0.05 vs. HCT-116 P (vehicle) or HT-29 P (vehicle); ^##^ *p* < 0.01 vs. HCT-116 P (vehicle) or HT-29 P (vehicle); ^###^ *p* < 0.001 vs. HCT-116 P (vehicle) or HT-29 P (vehicle).

Next, we studied cell growth and viability in cells after the overexpression of PARP-1 and subsequent inhibition of NO production (Figure 6A). The overexpression of PARP-1 led to increased growth in HCT-116 P while reducing it in HT-29 P, versus non-treated HCT-116 V and HT-29 V, respectively. Treatment with 20 µM 1400 W or siRNA2 restored cell growth to values similar to those found in non-treated control cells in both cases.

We also conducted a clonogenic assay for up to 10 days to evaluate the long-term effects of PARP-1 overexpression and the subsequent treatment with 1400 W or siNOS2 (Figure 6B). The results obtained were almost identical to those found in the MTT assay.

In addition to the effects on cell growth, we analyzed the induced cell death by apoptosis using Annexin V and propidium iodide and analyzing the stained cell populations by flow cytometry (Figure 6C). In HCT-116 V, the treatment with 1400 W or siNOS2 did not induce changes in apoptosis. However, we found increased cell death in HCT-116 P cells after treatment with siNOS2. Interestingly, the overexpression of PARP-1 induced an increase in cell death by apoptosis in HT-29 P cells. Treatment with 1400 W and siNOS2 induced opposite effects in HT-29 V and HT-29 P cells, increasing cell death in HT-29 V while decreasing it in HT-29 P (Figure 6C).

### 3.4. Inhibition of NO Production by iNOS Inhibited PARP-1 Effects on Stemness in CRC In Vitro

**Figure 7:**
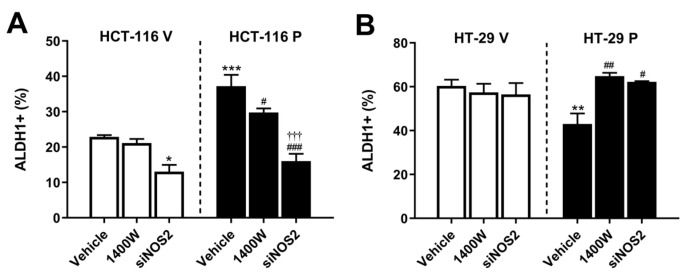
Percentage of ALDH1+ cells in (**A**) HCT-116 and (**B**) HT-29 in control cells (HCT-116 V and HT-29 V) and after overexpression of PARP-1 (HCT-116 P and HT-29 P) and, in all cases, without treatment (vehicle), treated with 20 µM 1400 W, or transfected with siNOS2. Data represent the mean ± SD. * *p* < 0.05 vs. HCT-116 V (vehicle); ** *p* < 0.01 vs. HT-29 V (vehicle); *** *p* < 0.001 vs. HCT-116 V (vehicle); ^#^ *p* < 0.05 vs. HCT-116 P (vehicle) or HT-29 P (vehicle); ^##^ *p* < 0.01 vs. HCT-116 P (vehicle); ^###^ *p* < 0.001 vs. HCT-116 P (vehicle); ^†††^ *p* < 0.001 vs. HCT-116 P (1400 W).

In order to investigate whether NO synthesized from iNOS acts as an intermediate in the regulation of stem cell properties by PARP-1 on CRC, we first analyzed the percentage of the subpopulation with positive aldehyde dehydrogenase 1 activity (ALDH1+). As expected, the overexpression of PARP-1 in HCT-116 cells (HCT-116 P) increased the ALDH1+ sub-population. Transfection with siNOS2 restored the levels of this sub-population to those found in the control transfected cells (HCT-116 V). In this case, the treatment of HCT-116 P cells with 20 µM 1400 W significantly decreased the ALDH1+ subpopulation, although it was less effective than the genic silencing of iNOS (Figure 7A). The transfection of HCT-116 V cells with siNOS2 also induced a decrease in the ALDH1+ sub-population (Figure 7A). On the other hand, the overexpression of PARP-1 in HT-29 cells (HT-29 P) led to a decrease in the percentage of ALDH1+ cells, and treatment with 1400 W or siNOS2 restored its level (Figure 7B). Representative plots of every condition analyzed are summarized in Figure S1.

**Figure 8:**
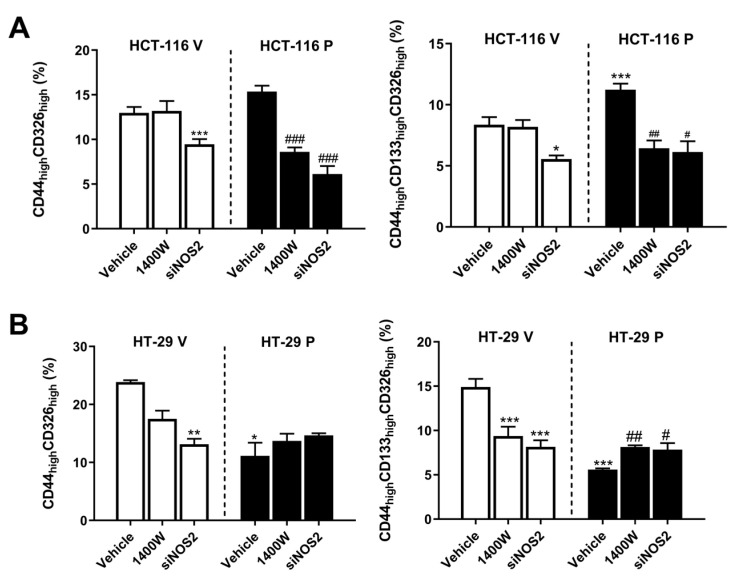
Percentage of CD44_high_CD326_high_ and CD44_high_ and CD326_high_CD133_high_ subpopulations in (**A**) HCT-116 and (**B**) HT-29 in control cells (HCT-116 V and HT-29 V) and after overexpression of PARP-1 (HCT-116 P and HT-29 P) and, in all cases, without treatment (vehicle), treated with 20 µM 1400 W, or transfected with siNOS2. Data represent the mean ± SD. * *p* < 0.05 vs. HCT-116 V (vehicle) or HT-29 V (vehicle); ** *p* < 0.01 vs. HT-29 V (vehicle); *** *p* < 0.001 vs. HCT-116 V (vehicle) or HT-29 V (vehicle); ^#^ *p* < 0.05 vs. HCT-116 P (vehicle) or HT-29 P (vehicle); ^##^ *p* < 0.01 vs. HCT-116 P (vehicle); ^###^ *p* < 0.001 vs. HCT-116 P (vehicle) or HT-29 P (Vehicle).

To characterize the phenotype of CSCs after the overexpression of PARP-1, and before and after treatments, we analyzed the percentage of cells with a high expression of CD44 and CD326 (CD44_high_CD326_high_) and with a high expression of CD44, CD326, and CD133 (CD44_high_CD326_high_CD133_high_), all of which are recognized as surface markers of the CSC subpopulation in CRC [5]. The percentage of the CD44_high_CD326_high_CD133_high_ subpopulation increased in HCT-116 cells overexpressing PARP-1 (HCT-116 P) versus the control transfected cells (HCT-116 V). Treatment with 20 µM of 1400 W or transfection with siNOS2 decreased this subpopulation in HCT-116 P to levels found in HCT-116 V cells (Figure 8B). The percentage of CD44_high_CD326_high_ cells did not change in HCT-116 P versus HCT-116 V, although treatment with 20 µM of 1400 W or transfection with siNOS2 significantly decreased it in HCT-116 P versus non-treated cells (HCT-116 P, vehicle) (Figure 8A). The percentage of double- or triple-marked cells in HCT-116 V cells decreased only after transfection with siNOS2 (Figure 8A).

These results indicate that the overexpression of PARP-1 transformed the HCT-116 cells towards a more aggressive phenotype, which is counteracted by either chemical or genic inhibition of NO release. This effect was also found in the control cells (HCT-116 V), but only after the genic inhibition of NO synthesis.

The overexpression of PARP-1 in HT-29 cells (HT-29 P) decreased both the CD44_high_CD326_high_ and CD44_high_CD326_high_CD133_high_ subpopulations versus control cells (HT-29 V), and the inhibition of NO release by iNOS only partially restored the CD44_high_CD326_high_CD133_high_ percentage (Figure 8B). Interestingly, in the HT-29 V cells, the inhibition of transcription of iNOS by siNOS2 decreased both the CD44_high_CD326_high_ and CD44_high_CD326_high_CD133_high_ subpopulations, and treatment with 1400 W only decreased the percentage of the CD44_high_CD326_high_CD133_high_ subpopulation (Figure 8B).

Contrary to the HCT-116 cells, the high expression of PARP-1 in HT-29 led to a less aggressive phenotype of CSCs, and their inhibition of NO production restored it significantly. It should be noted that in the HT-29 control cells, the effect was opposite, since the inhibition of iNOS/NO resulted in a cell culture with a lower percentage of CD44_high_CD326_high_ and CD44_high_CD326_high_CD133_high_ subpopulations.

In conclusion, and contrary to what happened in the HCT-116 cells, the overexpression of PARP-1 led to a less aggressive phenotype, was counteracted by either chemical or genic inhibition of NO release in HT-29.

**Figure 9:**
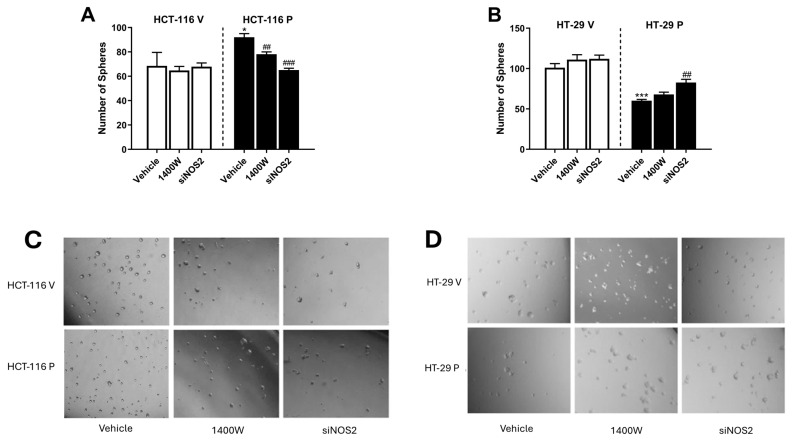
Number of spheres formed in (**A**) HCT-116 and (**B**) HT-29 in control cells (HCT-116 V and HT-29 V) and after overexpression of PARP-1 (HCT-116 P and HT-29 P) and, in all cases, without treatment (vehicle), treated with 20 µM 1400 W or transfected with siNOS2. Data represent the mean ± SD of three experiments performed in triplicate. * *p* < 0.05 vs. HCT-116 V (vehicle) or HT-29 V (vehicle); *** *p* < 0.001 vs. HT-29 V (vehicle); ^##^ *p* < 0.01 vs. HCT-116 P (vehicle) or HT-29 P (vehicle); ^###^ *p* < 0.001 vs. HCT-116 P (vehicle). Representative images of spheres obtained from (**C**) HCT-116-derived cells and from (**D**) HT-29-derived cells, before and after treatments.

Finally, we analyzed the anchorage-independent growth of the cells under free-serum conditions to determine the self-renewal capacity of cells. The overexpression of PARP-1 in HCT-116 led to an increase in the sphere-forming capacity. The inhibition of NO release by iNOS after treatment with 1400 W or transfection with siNOS2 significantly decreased the number of spheres formed. Conversely, the sphere-forming capacity decreased after PARP-1 overexpression in HT-29 and only transfection with siNOS2 almost restored the number of spheres formed to that found in the control cells (Figure 9).

## 4. Discussion

PARP1 is overexpressed in some types of tumors, including CRC, where it regulates important hallmarks of cancer [34]. Although it participates in other cellular functions, PARP-1 plays an essential role in DNA damage repair. As a consequence, its inhibition can cause cell death due to the accumulation of double-stranded DNA breaks (DSBs), mainly in cases with homologous recombination deficiency (HRD), for example when BRCA1 or BRCA2 mutations are present [35]. This phenomenon, called synthetic lethality, prompted clinical trials in which PARP-1 inhibitors were used as monotherapy in patients with germline BRCA-mutated ovarian or breast cancers [36,37]. In this sense, the lack of effectiveness found in CRC and other cancers was attributed to the low frequency of mutations in the homologous recombination (HR) system. Instead of this, an association between wild-type TP53 function and PARP inhibitor sensitivity was recently described using in vitro studies on cell lines of CRC [38]. TP53-mediated suppression of RAD51, an essential player in HR, seems to be a mechanism through which PARP inhibitors could act [38]. This is in accordance with other results previously published by our group, in which we pointed out the need to analyze the status of p53 before considering the use of PARP-1 inhibitors for the treatment of CRC [14]. In this study, we found that the overexpression of PARP-1 in CRC induced increased overall survival and disease-free survival in cases harboring a mutated p53, and that overexpression of PARP-1 is an independent prognostic factor for survival in those patients. These results led us to question treatment with PARP-1 inhibitors in these cases [14]. Interestingly, we also found that an overexpression of PARP-1 in CRC regulates the characteristics of CSCs in a p53-dependent manner, increasing their quantity and renovation capacity for wild-type p53 but inducing the opposite effects when p53 is mutated, which could explain the results found in patients [14].

PARP-1 is an important regulator of stemness in physiological and pathological conditions. In cancer, most studies involve the use of PARP inhibitors as therapeutic agents for analyzing CSC phenotypes and tumorigenic characteristics after treatments. However, the mechanism involved is not entirely elucidated [11,39]. In this work, we propose a mediating effect of the iNOS/NO system in CRC. Reciprocal regulation of PARP-1 and iNOS has been described in several pathological conditions, including inflammation, as a protective mechanism [21,22,40]. According to this, in our study, the overexpression of PARP-1 rendered an increased expression of iNOS followed by increased NO release, while the inhibition of iNOS activity induced a decrease in PARP-1 expression regardless of p53 status, and also reversed the effect that the overexpression of PARP-1 had on the growth and stem characteristics in the in vitro model used. These results, together with the fact that both proteins are co-expressed in cells, lead us to propose that the NO produced by iNOS could act as an intermediary in the process of CSC regulation by PARP-1 in CRC.

Cell death by apoptosis is usually accompanied by an increased activity of caspases. Both caspase-3 and caspase-7 cleave PARP-1 into two fragments of 89 kDa and 24 kDa, resulting in its inactivation. In fact, the 89-kD fragment containing the automodification and catalytic domains of the enzyme has a reduced DNA-binding ability [41,42]. Genetic silencing with siNOS2 led to an increase in apoptosis in HCT-116 cells overexpressing PARP-1. However, the levels of cleaved PARP-1 are similar in all conditions, indicating that the reduction in PARP-1 expression found is due to transcriptional regulation by NO rather than to caspase cleavage.

Cytoplasmic localization of PARP-1 has been described in cancer cells, although its functions are still unclear [43]. In pancreatic cancer, it promotes cancer tumorigenesis and resistance to therapy [44]. Similarly, in breast cancer patients, a high cytoplasmatic expression of PARP-1 correlates with aggressivity and predicts sensitivity to chemotherapy and prognosis [45]. These studies and others [41,42] identified cytoplasmatic PARP-1 in the 89 kDa cleaved PARP-1 form. In the in vitro study, we observed both nuclear and cytoplasmatic PARP-1 staining in cells. While the nuclear localization of PARP-1 changes after overexpression of the protein and subsequent treatment with 1400 W, at least in HT-29 cells, cytoplasmatic PARP-1 was similar under the conditions analyzed, showing its lack of relevance in this study.

As a highly reactive molecule, NO can induce DNA damage, which has been linked with inflammation-associated carcinogenesis [24]. In these cases, NO induces p53 accumulation and p53-induced cell cycle arrest and apoptosis [46,47]. It has also been reported that p53 can bind to the iNOS promoter and inhibit its transcription [48]. Overall, this could lead to a selection pressure for cells expressing a mutant p53 and could also lower the expression of iNOS in wild-type p53 tumors compared to those with mutant p53, in accordance with previous reports [49,50]. However, we did not find differences in iNOS expression regarding p53 status in our study cohort. On the contrary, in the in vitro model, we found a lower basal expression of iNOS in the cell line with mutated p53 than in the cell line with wild-type p53, as previously reported [26]. This could be a consequence of the different mutational background between them. In fact, iNOS expression can be affected not only by p53, but also by other frequently mutated oncogenes in CRC, such as APC [51] or KRAS [52]. On the other hand, and following NO-induced DNA damage, PARP-1 can be activated [47]. Nevertheless, only 1–2% of diagnosed CRCs come from a chronic inflammatory disease [53], and mechanisms of PARP-1 activation that are not associated with damage to cellular DNA have also been described [54,55]. In these cases, and after PARP-1 activation, iNOS expression and activity may also increase [22]. Accordingly, we found increased iNOS expression after PARP-1 overexpression in our in vitro model, regardless of p53 status, indicating that the basal expression of iNOS in cells has no implications for this PARP-1 effect. Overall, this could explain the correlation found between PARP-1 and iNOS expressions in all cases, regardless of the p53 status in the cohort of patients included in the study.

In the patients, iNOS expression increased in poorly differentiated tumors harboring a wild-type p53. Since dedifferentiation has been linked with the presence of CSCs [5], we analyzed the expression of iNOS regarding both the expression of CSC markers and p53 status. High levels of CSC markers imply high levels of iNOS expression, although only in wild-type p53 tumors. These results are similar to those we previously reported for PARP-1 [14] and to those found in the subcohort used in this study. Interestingly, the expression of iNOS and PARP-1 correlated, regardless of the levels of expression of CSC markers and the status of p53. In the in vitro model, the overexpression of PARP-1 in the HCT-116 cells carrying a wild-type p53 conferred a greater growth capacity, associated with a higher content of CSCs with a more aggressive phenotype and with a greater capacity for self-renewal, while the opposite effects were found in HT-29 cells, as expected [14]. The selective inhibition of iNOS expression and activity reverted all these effects in both cell lines. These results imply that iNOS could act as a mediator of the differential regulation of CSCs by PARP-1, depending on p53.

Even though the current study does not analyze the mechanism behind the role of iNOS inhibition in the characteristics of colorectal CSCs, previous results have pointed to the potential involvement of the Wnt/β-catenin Notch pathways, as well as the transcription factor NF-κβ [29,56]. In mouse models of carcinogenesis, it was reported that NF-κB activation increased Wnt/β-catenin signaling activity and induced the dedifferentiation of non-stem cells that acquired tumor-initiating capacity [57]. PARP-1 interacts with NF-κB to regulate cell growth and apoptosis, as well as epithelial to mesenchymal transition [58]. On the other hand, treatment with PARP-1 inhibitors regulates β-catenin signaling and resistance to chemotherapy [11]. Thus, it is possible that PARP-1 regulates stemness through iNOS/NO, which in turn could modulate NF-κB/Wnt/β-catenin signaling in CRC. However, more research is warranted to confirm this hypothesis. Depending on the cell type, Notch signaling can play oncogenic or tumor-suppressor roles. In CRC, activation of the Notch pathway increases CSC subpopulations [5]. Interaction between HES1, a downstream effector of Notch, and PARP-1 has been identified to finally induce apoptosis in B leukemic cells, as a protective mechanism against this type of cancer [59]. Nevertheless, it is still not known whether PARP-1 regulates Notch signaling and its effects on cell dedifferentiation in CRC.

Despite the lack of an in-depth analysis of the mechanism by which PARP-1 differentially regulates the characteristics of CSCs in CRC according to the state of p53, our results clarify, at least in part, the dual effect of NO on cancer described in the literature [30,31,32]. In terms of PARP-1 overexpression, NO induces tumor progression in cases with wild-type p53, exerting the opposite effect when p53 is mutated.

However, more complex research using models closer to a real tumor are needed to further elucidate how PARP-1 expression influences the effect of NO/iNOS on CSC characteristics. This consideration comes from the fact that NO can be released from a cancer cell itself [27,60,61] and from cells in the microenvironment [56,62,63], i.e., fibroblasts, endothelial and/or immune cells [27]. There are other isoforms of NOS, called neuronal (nNOS) and endothelial (eNOS) NOS, initially associated with physiological signaling in the brain and blood vessels, respectively [23]. The isoform responsible for NO release in the tumor microenvironment could depend on the cell of origin. Thus, iNOS would be the main isoform in immune cells and fibroblasts, while eNOS would be expressed mainly in endothelial cells [27]. On the other hand, CSC-produced NO is secreted in the tumor microenvironment, inducing a wide range of phenotypic changes in stromal cells, which have been implicated in drug resistance, invasion, and metastasis [27]. Finally, although the iNOS isoform has received most of the attention, the recent literature indicates that the endothelial isoenzyme (eNOS) can also be present in cancer cells, where it can modulate the phenotype of CSCs, as reported for CRC [56] and prostate cancer [64], which adds more complexity to the relationship between NO and cancer.

## 5. Conclusions

Taking into account all of the above and given that the use of PARP-1 inhibitors has been linked to resistance after a period of treatment, the use of selective inhibitors of iNOS could be considered in cases of high PARP-1 expression and also those harboring wild-type p53 in CRC. However, more research is needed to uncover the pathways implicated. In addition, it would be necessary to expand this research by using multicellular 3D models that include both stem and non-stem cancer cells, as well as cells from the microenvironment, since NO can be synthetized by different cells in the tumor. Finally, both PARP-1 and iNOS have been implicated in the regulation of stemness in several types of cancer. In consequence, it would be interesting to study this regulatory pathway in them, the results of which could be translated into testing therapies based on selective iNOS inhibitors.

## Figures and Tables

**Table 1:** Relationship between iNOS expression and the clinicopathological characteristics of the patients included in the study.

Characteristic		All ^1^	*p*	wtp53 ^2^	*p*	mtp53 ^3^	*p*
		Median ± IQR ^5^		Median ± IQR ^5^		Median ± IQR ^5^	
Age (y) *^,4^	<72	1.44 (0.41–3.49)	0.978	1.68 (0.41–4.88)	0.348	1.15 (0.28–2.59)	0.331
	≥72	1.10 (0.33–3.49)		1.41 (0.39–3.55)		0.87 (0.19–2.30)	
Gender *	Male	1.41 (0.41–3.29)	0.827	1.63 (0.41–3.98)	0.631	1.14 (0.37–2.82)	0.281
	Female	1.22 (0.29–3.64)		0.80 (0.29–4.33)		0.96 (0.17–1.96)	
Location *	Colon	1.35 (0.40–3.50)	0.541	1.63 (0.39–4.82)	0.342	1.02 (0.27–2.31)	0.957
	Rectum	1.01 (0.40–3.29)		0.67 (0.54–2.89)		1.01 (0.10–13.17)	
DG ^†,&^	Well	1.06 (0.41–2.62)	0.189	1.10 (0.42–2.74)	0.020	1.03 (0.39–3.04)	0.485
	Moderately	1.17 (0.38–3.16)		1.76 (0.39–5.20)		0.96 (0.17–1.63)	
	Poor	2.82 (0.41–6.65)		2.39 (0.38–6.41)		1.63 (0.34–16.23)	
T stage	T1 + T2	1.37 (0.43–7.08)	0.034	5.32 (0.19–13.10)	0.004	0.92 (0.43–5.04)	0.079
	T3	1.03 (0.27–3.12)		1.37 (0.40–3.25)		0.93 (0.17–2.31)	
	T4	1.76 (1.04–5.41)		2.04 (0.59–7.41)		1.60 (1.29–4.77)	
LNM *^,#^	Absent	1.63 (0.44–3.64)	0.827	1.72 (0.44–3.37)	0.150	0.95 (0.22–2.24)	0.877
	Present	1.17 (0.37–3.25)		1.37 (0.33–7.20)		1.22 (0.35–3.49)	
pTNM Stage *	Stage I + II	1.41 (0.47–3.26)	0.408	1.80 (0.67–3.29)	0.562	0.95 (0.26–2.21)	0.526
	Stage III + IV	1.24 (0.37–3.70)		1.19 (0.31–5.56)		1.22 (0.24–3.5)	

* Analysis was performed using non-parametric Mann–Whitney U test for independent samples or ^†^ Kruskal–Wallis test for independent samples; ^#^ lymph node metastasis; ^&^ differentiation grade; ^1^ all cases studied; ^2^ p53 wild-type tumors; ^3^ p53 mutated tumors; ^4^ dichotomized by the median; ^5^ interquartile range.

**Table 2:** Correlation of iNOS and PARP-1 expression in tumors from CRC patients, considering the status of p53.

	All ^1^	wtp53 ^2^	mtp53 ^3^
Rs	0.419	0.342	0.360
*p* ^4^	0.0001	0.002	<0.0001

^1^ All cases studied; ^2^ p53 wild-type tumors; ^3^ p53 mutated tumors; ^4^ Pearson’s correlation coefficient.

**Table 3:** Correlation of iNOS and PARP-1 expressions in tumors from CRC patients, considering the status of p53 and the expression of both CD44 and CD133 markers.

	wtp53 ^2^		mtp53 ^3^	
	CD44CD133		CD44CD133	
	Low	High	Low	High
Rp ^1^	0.343	0.306	0.633	0.604
*p*	0.040	0.064	<0.0001	0.029

^1^ Pearson’s correlation coefficient; ^2^ p53 wild-type tumors; ^3^ p53 mutated tumors.

## Data Availability

The original contributions presented in this study are included in the article/Supplementary Materials. Further inquiries can be directed to the corresponding author.

## Supplementary Materials

The following supporting information can be downloaded at https://www.mdpi.com/article/10.3390/biom15010125/s1, Figure S1: Representative plots of the ALDH1 assay; Table S1: Characteristics of the patients include in the study; Figure S2: Original Western Bolt images of Figure 4A; Table S2: Primers used to analyze P53 mutations; Table S3: Primers used to determine PARP-1, iNOS, CD44, CD133, UBC, TBP, and RPS13 expression; Table S4: iNOS and PARP-1 expression in tumor and non-tumoral samples from all patients and patients stratified by p53 status in wild-type (wtp53) and mutated (mtp53); Table S5: Relationship between PARP-1 expression and clinicopathological characteristics of the patients included in the study.
