# Supplementary material for: iNOS-Produced Nitric Oxide from Cancer Cells as an Intermediate of Stemness Regulation by PARP-1 in Colorectal Cancer
Source: Biomolecules. 2025 Jan 14;15(1):125. doi: 10.3390/biom15010125 (PMC11763104; doi:10.3390/biom15010125)
Supplement: Supplementary file 1 [file biomolecules-15-00125-s001.zip › biomolecules-3405732-supplementary.pdf]

Supplementary Materials

Table S1. Characteristics of the patients include in the study

Characteristics	N (%)
**Gender**	
Female	73 (38.8)
Male	115 (71.2)
**Location**	
Colon	176 (93.6)
Rectum	12 (6.4)
**Histological Type**	
Well differentiated	45 (25.0)
Moderately differentiated	106 (58.9)
Poorly differentiated	29 (16.1)
**T stage**	
T1	2 (1.1)
T2	24 (12.8)
T3	127 (67.9)
T4	34 (18.2)
**pTMN Stage**	
Stage I	22 (11.8)
Stage II	74 (39.8)
Stage III	75 (40.3)
Stage IV	15 (9.1)
**Nº of lymph nodes removed**	
<12	71 (35.5)
≥12	130 (64.5)

Table S2. Primers used to analyze P53 mutations

TP53	Forward	Reverse
Exon 2-4	agctgtctcagacactggcatggtgttgg	cactgacaggaagccaaagggtgaagagg
Exon 5-6	gttgctttatctgttcacttgtgccctgac	tagggaggtcaatatagcagcaggagaaag
Exon 7-9	cagcctgggcgacagagcgagattccatc	aaccaggagccattgtctttgaggcatcac
Exon 10	tacttgaagtgcagtttctactaaatgcatg	aggaagactaaaaaaatgtctgtgcagggc

Table S3. Primers used to determine PARP-1, iNOS, CD44, CD133, UBC, TBP and RPS13 expression

Gene	Forward	Reverse
PARP-1	agggcaagcacagtgtcaaa	tacccatcagcaacttagcg
iNOS	gatgaggaccacatctaccaggag	atagcgcttctggctcttgagctg
CD44	gctttcaatagcaccttgcccacaatgg	aaagaggtcctgtcctgtccaaatcttcc
CD133	tccacagaaatttacctacattgg	cagcagagagcagatgacca
UBC	tgggatgcaaatcttcgtgaagaccctgac	accaagtgcagagtggactctttctggatg
TBP	ttgtccttttttgccatttgctgggctcctc	gtcatcagtggagacggtttccatttaacc
RPS13	ggtgttgcacaagtacgttttgtgacaggc	tcatatttccaattgggagggaggactcgc

Table S4. iNOS and PARP-1 expression in tumour and non-tumoral samples from all patients and patients stratified by p53 status in wild-type (wtp53) and mutated (mtp53).

		All cases		Wtp53		Mtp53	
		Median ± CL[1]	P[2]		P[2]		P[2]
**iNOS**	Tumor	180 (104-266)	<0.0001	44 (14-116)	0.006	37 (13-81)	ns
	Non-tumor	72 (52-483)		27 (13-49)		30 (12-75)	
**PARP-1**	Tumor	985 (1103-4936)	<0.0001	878 (376-1643)	0.001	770 (416-1775)	0.013
	Non-tumor	876 (791-5538)		476 (141-1198)		686 (207-1181)	

[1] CL: confidence limits; Comparisons were performed using a non-parametric test for two related samples.

Table S5. Relationship between PARP-1 expression and clinicopathological characteristics of the patients included in the study

Characteristic		All[1] Median ± CL[5]	P	wtp53[2] Median ± CL[5]	P	mtp53[3] Median ± CL[5]	P
**Age (y)**[*4]	<72	1.41 (0.92-2.34)	0.825	1.41 (0.99-2.10)	0.763	1.06 (0.81-1.72)	0.588
	≥72	1.56 (0.84-2.25)		1.33 (0.96-2.10)		1.70 (0.89-1.76)	
**Gender**[*]	Male	1.54 (0.91-2.63)	0.245	1.40 (0.94-2.46)	0.698	1.36 (0.81-2.33)	0.250
	Female	1.38 (0.83-2.11)		1.42 (0.96-2.04)		1.25 (0.9-1.76)	
**Location**[*]	Colon	1.50 (0.89-2.25)	0.954	1.33 (0.94-2.05)	0.985	1.23 (0.81-2.33)	0.514
	Rectum	1.41 (0.94-5.06)		1.56 (1.01-5.06)		1.66 (0.10-9.05)	
**DG**[†&]	Well	1.17 (0.64-2.32)	**0.030**	1.10 (0.64-1.27)	**0.007**	1.20 (0.68-2.96)	0.934
	Moderately	1.63 (1.00-2.34)		1.70 (1.05-2.69)		1.36 (0.84-1.69)	
	Poor	1.60 (0.99-1.77)		1.60 (1.05-1.76)		1.45 (0.99-3.61)	
**T stage**	T1+T2	1.73 (0.88-2.88)	0.109	1.47 (1.13-2.71)	0.075	1.93 (0.66-10.88)	0.426
	T3	1.48 (0.89-2.24)		1.54 (0.87-2.27)		1.23 (0.85-2.39)	
	T4	1.90 (1.23-2.76)		2.10 (1.62-4.65)		1.75 (1.22-3.35)	
**LNM**[*#]	Absent	1.58 (0.82-2.34)	0.823	1.26 (1.01-2.46)	0.823	1.63 (0.79-2.31)	0.920
	Present	1.46 (0.92-2.04)		1.54 (0.92-2.02)		1.19 (0.87-2.14)	
**pTNM Stage**[*]	Stage I+II	1.40 (0.81-2.19)	0.838	1.23 (0.88-2.21)	1.000	1.63 (0.78-2.18)	0.713
	Stage III+IV	1.52 (0.93-2.29)		1.56 (0.96-2.04)		1.16 (0.86-2.34)	

*Analysis was performed using non-parametric Mann-Whitney U test for independent samples or †Kruskal-Wallis test for independent samples; #Lymph Node Metastasis; [&]Differentiation Grade; [1]All cases studied; [2] p53 wild-type tumours; [3]p53 mutated tumours; [4]Dichotomized by the median; [5]Confident limits.

	HCT-116 V		HCT-116 P		HT-29 V		**HT-29 P**	
	+ DEAB	-DEAB	+ DEAB	-DEAB	+ DEAB	-DEAB	+ DEAB	-DEAB
Vehicle	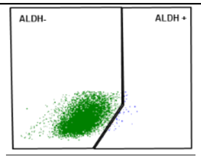	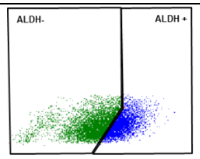	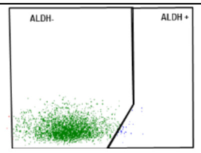	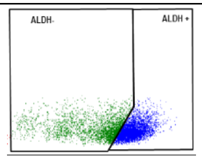	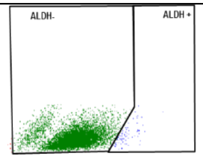	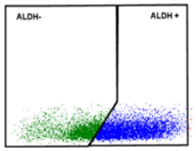	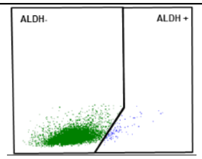	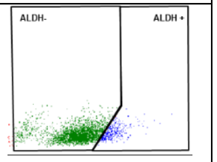
1400W		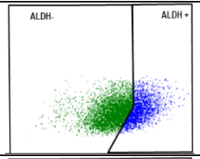		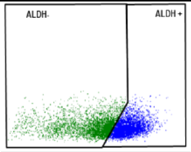		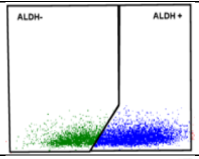		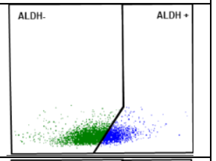
siNOS2		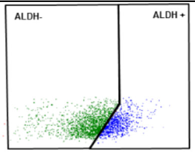		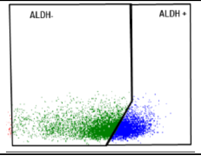		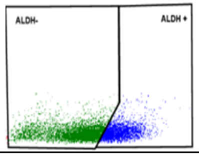		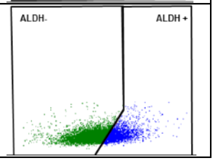

	HCT-116 V		HCT-116 P		HT-29 V		**HT-29 P**	
	+ DEAB	-DEAB	+ DEAB	-DEAB	+ DEAB	-DEAB	+ DEAB	-DEAB
Vehicle	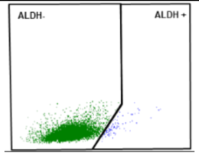	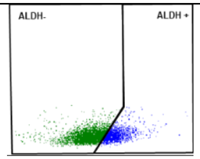	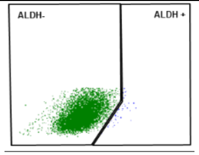	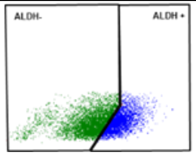	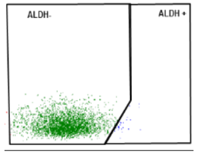	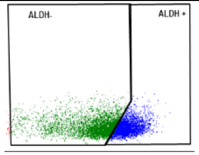	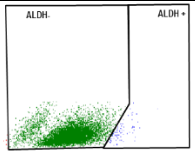	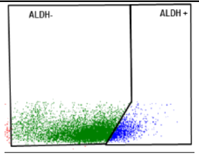
1400W		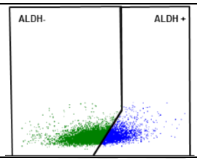		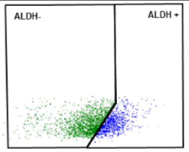		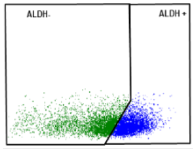		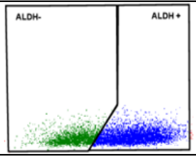
siNOS2		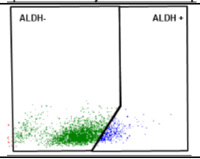		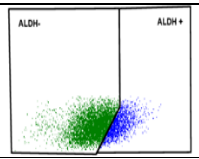		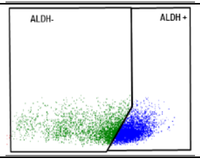		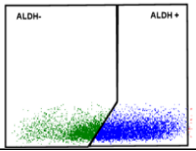

Figure S1. Representative plots obtained by flow cytometry of ALDH1+ activity cells in every condition studied.
