# Supplementary figures and images for: iNOS-Produced Nitric Oxide from Cancer Cells as an Intermediate of Stemness Regulation by PARP-1 in Colorectal Cancer
Source: Biomolecules. 2025 Jan 14;15(1):125. doi: 10.3390/biom15010125 (PMC11763104; doi:10.3390/biom15010125)

HCT-116

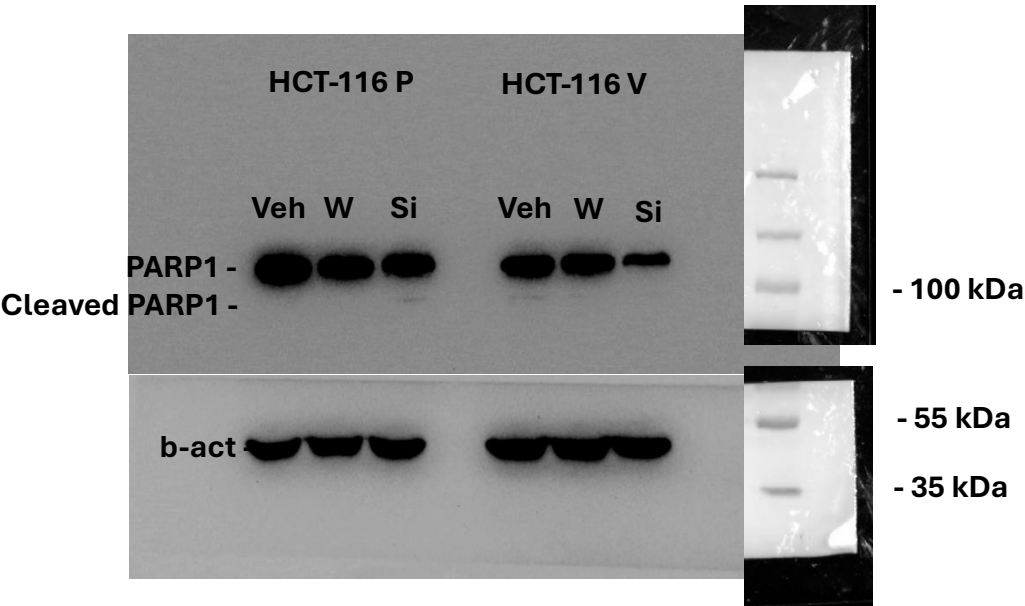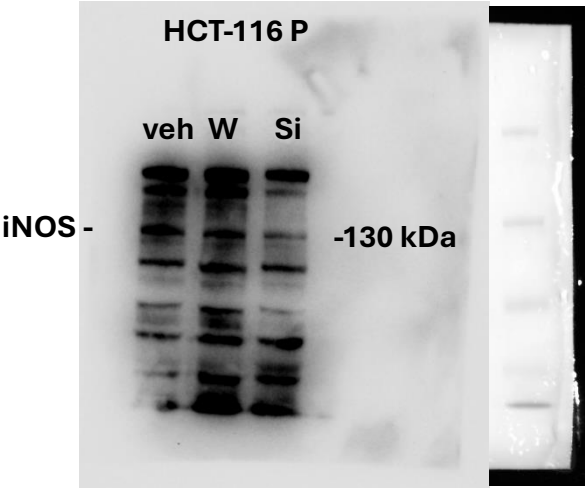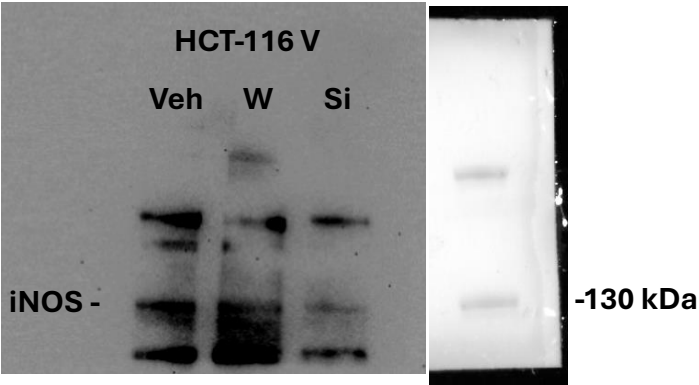

HT-29

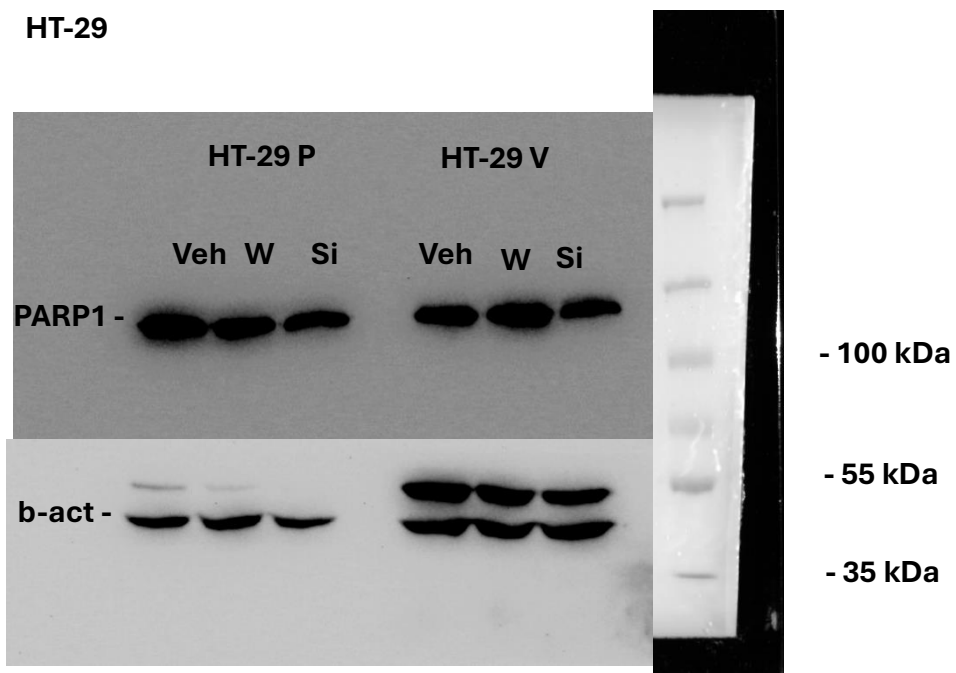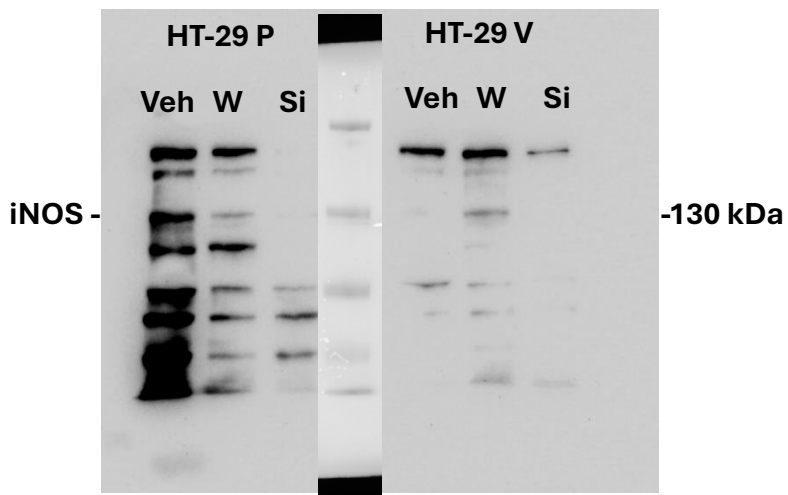

Figure S2. Original Western Bot images of Figure 4A.

Supplement: Supplementary file 1 [file biomolecules-15-00125-s001.zip › Figure S2.pdf]
